# The translation, validity and reliability of the German version of the Fremantle Back Awareness Questionnaire

**DOI:** 10.1371/journal.pone.0205244

**Published:** 2018-10-04

**Authors:** Katja Ehrenbrusthoff, Cormac G. Ryan, Christian Grüneberg, Benedict M. Wand, Denis J. Martin

**Affiliations:** 1 Health and Social Care Institute, Teesside University, Middlesbrough, Tees Valley, United Kingdom; 2 Hochschule für Gesundheit, Department of Applied Health Sciences, Bochum, Germany; 3 School of Physiotherapy, The University of Notre Dame Australia, Fremantle, Western Australia, Australia; HES-SO Valais-Wallis, SWITZERLAND

## Abstract

**Background:**

The Fremantle Back Awareness Questionnaire (FreBAQ) claims to assess disrupted self-perception of the back. The aim of this study was to develop a German version of the FreBAQ (FreBAQ-G) and assess its test-retest reliability, its known-groups validity and its convergent validity with another purported measure of back perception.

**Methods:**

The FreBaQ-G was translated following international guidelines for the transcultural adaptation of questionnaires. Thirty-five patients with non-specific CLBP and 48 healthy participants were recruited. Assessor one administered the FreBAQ-G to each patient with CLBP on two separate days to quantify intra-observer reliability. Assessor two administered the FreBaQ-G to each patient on day 1. The scores were compared to those obtained by assessor one on day 1 to assess inter-observer reliability. Known-groups validity was quantified by comparing the FreBAQ-G score between patients and healthy controls. To assess convergent validity, patient’s FreBAQ-G scores were correlated to their two-point discrimination (TPD) scores.

**Results:**

Intra- and Inter-observer reliability were both moderate with ICC_3.1_ = 0.88 (95%CI: 0.77 to 0.94) and 0.89 (95%CI: 0.79 to 0.94), respectively. Intra- and inter-observer limits of agreement (LoA) were 6.2 (95%CI: 5.0–8.1) and 6.0 (4.8–7.8), respectively. The adjusted mean difference between patients and controls was 5.4 (95%CI: 3.0 to 7.8, p<0.01). Patient’s FreBAQ-G scores were not associated with TPD thresholds (Pearson’s *r* = -0.05, p = 0.79).

**Conclusions:**

The FreBAQ-G demonstrated a degree of reliability and known-groups validity. Interpretation of patient level data should be performed with caution because the LoA were substantial. It did not demonstrate convergent validity against TPD. Floor effects of some items of the FreBAQ-G may have influenced the validity and reliability results. The clinimetric properties of the FreBAQ-G require further investigation as a simple measure of disrupted self-perception of the back before firm recommendations on its use can be made.

## Introduction

Low back pain is a major cause of disability worldwide [1] and is associated with substantial health care costs [2]. Many interventions attempt to normalise assumed peripheral structural pathology [3]. However, current treatment strategies provide limited and short-term pain relief [4].

The cortical body representation is distorted in people with persistent pain which may play an important role in the development and/or maintenance of pain [5]. Early work by Flor et al [6] using brain imaging identified somatosensory disorganization in patients with chronic low back pain (CLBP). More recent imaging studies in people with CLBP have shown structural and functional alterations in cortical and subcortical areas, associated with the processing of sensory information [7–9].

These changes can present clinically as alterations in a person’s body perception [10–13], decreased ability to distinguish/interpret peripheral sensory stimuli [14–16] and impaired lumbopelvic motor control [17]. Interventions targeting these perceptual distortions may present novel approaches to managing persistent low back pain [18–23]. Due to the growing research and clinical interest in cortical body representation, there is an increasing need for valid and reliable body perception measurement tools which are quick and easy to deliver clinically. A recent systematic review highlighted the lack of such measures and the need for further work in this area [24]

The Freemantle Back Awareness Questionnaire (FreBAQ) is a simple tool that claims to assess back-specific altered body perception [25]. It comprises 9 items with a five-point Likert scale attempting to investigate neglect-associated features, proprioceptive acuity, and a person’s perceived body image [26, 27].

In people with CLBP, the FreBAQ has been associated with a number of clinical characteristics such as pain duration (Pearson correlation ρ = 0.357, p = 0.01) and pain intensity (Spearman’s *rho* = 0.40, p = 0.004) [26] though others have found no such relationship [28]. As a measure of body perception it has demonstrated evidence for known-groups validity (median difference between healthy controls and people with CLBP = 11, Mann-Whitney test, p< 0.001) and reliability (ICC2,1_agreement_ = 0.652 (95% CI: 0.307 to 0.848),ICC2,1_consistency_ = 0.667 (95% CI: 0.317 to 0.857) [26] but did not provide evidence of convergent validity with other body perception measures in a different pain population [29]. The FreBAQ was recently translated and validated into Japanese (FreBAQ-J) [27] and Dutch [28]. However, a German version does not yet exist within the peer-reviewed literature.

The aim of this study was to produce a German version of the FreBAQ (FreBAQ-G) and assess its test-retest reliability, its known-groups validity and its convergent validity as a measure of disrupted self-perception of the back.

## Methods

### Translation and face validity

The FreBAQ translation was conducted following international guidelines for the transcultural adaptation of self-reported measures [30]. This process attempts to maintain content validity by ensuring that similar issues are covered, taking into consideration language differences and potentially differing sociocultural backgrounds [31].

Firstly, two native German speakers translated the original English FreBAQ independently of each other into German. One translator (K.E.) was a physiotherapist and researcher well acquainted with the subject area and one was a university graduated translator and occupational therapist but uninformed regarding the subject area (M.D.). Consensus regarding discrepancies was reached through discussion between both translators. Secondly, the revised German version was back-translated into English by two different translators, neither of whom had any specialist knowledge in the subject area of physiotherapy or chronic pain; one (J.J.H.) was a university lecturer in psychology and a native English speaker fluent in German while the other (A.J.) was a German English teacher, fluent in English. Again, consensus was reached regarding differences in wording through discussion between translators. Thirdly, the whole translation process was documented by K.E. and discussed with the developer of the original English version (B.W.) arriving at a pre-final German version (FreBAQ-G) (AppendixS1).

Once this translation process was complete, the FreBAQ-G was provided to a group of individuals with CLBP and healthy controls to assess its face validity. Face validity can be defined as “the degree to which a measurement instrument, looks as though it is an adequate reflection of the construct to be measured”[32]. As there are currently no standards concerning its measurement or quantification [33], three major aspects were assessed; completeness of content, comprehensibility and time to complete. Both groups provided feedback on completeness of content (“Do you think that this questionnaire covers the most important aspects of altered back related perception? [Yes/No]”; “If “NO” which aspects would you incorporate?”), comprehensibility (“Are the questions sufficiently comprehensibly worded? [Yes/No]”; “If “No” which items are not sufficiently comprehensible?”) and time to complete (“Is the time needed for filling in the questionnaire appropriate?) scored on a 0–10 scale with 0 representing “unacceptably long” and 10 “completely ok”). These questions were deemed to provide information on overall usability and whether it potentially needed revision due to content or linguistic ambiguities [28].

Floor and ceiling effects of the questionnaire were investigated in the CLBP group on an item level and by assessment of the total scores. Ceiling and floor effects occur when a considerable proportion of subjects score highest or lowest on a scale, demonstrating the measure unsuitable to discriminate between subjects at either extreme of the scale [34, 35]. Ceiling or floor effects were considered present if more than 15% of respondents achieved the highest or lowest possible score, respectively [36].

### Participants

35 patients with non-specific CLBP were recruited consecutively from physiotherapy practices in Bochum, Germany between June 2013 and December 2014. Participants had to meet the following inclusion criteria: age ≥18 years; non-specific CLBP with or without leg pain (for those with leg pain, the back pain had to be dominant); duration of symptoms ≥6 months; sufficient cognitive and German language ability to understand both oral and written instructions, provide feedback and informed consent. Participants were excluded if they were pregnant or less than 6 months post-partum, had signs and symptoms indicating serious spinal pathologies (i.e. red flags), thus differentiating them clinically from people with non-specific CLBP [37].

In addition, a sample of 48 healthy participants, recruited from staff and students (lower age limit: 18 years) at the University of Applied Health Sciences in Bochum, Germany were recruited. According to the original FreBAQ protocol by Wand et al [26] healthy participants had to meet the following criteria: currently back pain free, no episode of back pain within the last two years restricting them from work or leisure activities, sufficient cognitive and German language ability to understand both oral and written instructions, provide feedback and informed consent. Exclusion criteria were pregnancy or less than 6 months post-partum or significant spinal deformities. The study was approved by Teesside University’s School of Health and Social Care Research Governance and Ethics Board (Study No 186/12) and the Ethics committee of the German National Physiotherapists Society (Ethics committee submission number: 2013–02). Before study commencement, all participants provided written informed consent to participate in the study.

The patient population provided basic demographic data and clinical characteristics as well as a battery of outcome measures recommended for back pain research, including measures of symptom severity and frequency, physical function, general well-being and current work disability [38]. Demographic information comprised: age, sex, height, weight, body mass index (BMI), and current working status. Clinical characteristics comprised: duration of symptoms, FreBAQ-G, Brief Pain Inventory Short form (BPI) [39], (pain intensity and interference); Roland Morris Disability Questionnaire (RMDQ) [40] (function); Hospital Anxiety and Depression Scale (HADS) [40] (anxiety and depression) and Euroquol 5D-3L [41] (quality-of-life).

The control group provided the same basic demographic data and also completed the HADS and the FreBAQ-G. Regarding the FreBAQ-G instructions the wording was slightly adopted in that the phrase “other patients” was replaced by “other people” and the section concerning current pain experience was replaced by “please indicate to which degree your back feels like this”.

Within the data analysis a FreBAQ-G item which was not answered was categorised as ‘not endorsed’, in keeping with Wand et al [26] and scored as zero, representing “never feels like this”. All analyses were conducted using SPSS, version 24 (IBM, Armok, USA) or Microsoft Excel 2010, version 14 (Microsoft, Redmond, USA).

### Relationship to clinical status

To quantify the association between the FreBAQ-G and the clinical characteristics of the patients a series of Pearson’s or Spearman’s correlations were conducted dependent upon the normality of the data. An r value of 0.10, 0.30 and 0.5 represented small, medium and large correlations respectively [42]. We hypothesised that people scoring higher on the FreBAQ-G, indicating a more disturbed self-perception of the back, would achieve poorer scores on other clinical outcome measures, assessing different constructs, such as pain and physical function.

### Reliability

One assessor (KE) provided the FreBAQ-G to each participant with CLBP on two separate days. The participants were asked to complete the questionnaire independently in the presence of the assessor in a quiet room of the University’s outpatient department. The assessor did not provide any assistance with the completion of the questionnaire. Day 1 and day 2 were on average one week apart. FreBAQ-G scores between day 1 and day 2 (collected by assessor 1), were compared to quantify intra-observer reliability over one week. To quantify inter-observer reliability, a second assessor provided the questionnaire to each participant on day 1 approximately two hours after it was provided by KE, and this was compared to the scores obtained by the first assessor’s administration on day 1. Assessors were blind to previous FreBAQ-G scores, as the questionnaires were immediately filed in a folder and only analysed upon the participant’s completion of the study. On day 2, the participant was not provided with any information regarding their previous scores to reduce the risk of recall bias.

The data obtained by assessor one on day one was used to quantify the frequencies of responses per item. The systematic bias (mean (95% CI) between data collected from two assessors on the same day and from one assessor from two sessions was determined using a paired t-test. Within-subjects standard deviations, defined as the standard error of measurement, coefficients of variation, limits of agreement and a random-error only intraclass correlation coefficient (ICC), model 3.1, were calculated to quantify the random error component within and between assessors.

The within-subjects SD was then used within a statistical power calculation to estimate whether the random measurement error identified in this study was small enough to detect a clinically relevant change in FreBAQ scores with a feasible sample size. As no MCID for the FreBAQ exists a value of 10 was chosen as the MCID within the power calculation based upon previous data quantifying the difference in FreBAQ between people with back pain and healthy controls [26]. ICC_3.1_ scores of <0.75 were considered to demonstrate poor reliability, 0.75–0.89 moderate and ≥0.90 excellent reliability [43]. Statistical significance was set at p≤0.05 [44].

### Validity

#### Internal consistency

Internal consistency of the FreBAQ-G was assessed by calculating the Cronbach’s alpha coefficient. A correlation coefficient of at least 0.7 was defined as indicative of adequate inter-relatedness of items [33].

#### Convergent validity

Convergent validity is defined as a positive correlation between instruments assuming to measure the same underlying construct [33]. To assess the convergent validity the patient’s FreBAQ-G scores were correlated to their two-point discrimination (TPD) scores measured by assessor one on day 1. TPD is a simple clinical test of tactile acuity which measures the minimum distance between two points on the skin that can be obviously detected with smaller distances indicating better acuity [45]. It has been shown to be a valid measure of cortical reorganization when compared against the gold standard measure of fMRI [46] and other clinical tests which purport to measure body awareness indirectly such as movement control tests [47]. The TPD collection method and data have been published previously [48]. The measurement tool was a two-point discrimination caliper (Nexgen Medical Systems, Florida,USA) with a 1 mm precision. To minimise the risk of assessor bias assessor 1 was not aware of the FreBAQ results when undertaking the TPD assessment. In principal, we hypothesised a positive correlation between FreBAQ total scores and TPD results. The direction of this hypothesis was based on findings from previous studies, demonstrating an association between an altered body image and tactile acuity, measured by TPD [14, 16, 23].

#### Known-groups validity

Known-groups validity is defined as an instrument’s ability to differentiate between individuals with a specific condition and healthy individuals [33] or its ability to differentiate between two groups on a construct on which they theoretically should differ [49]. To investigate the known-groups validity of the FreBAQ-G the total score on the questionnaire was compared between the group of patients and healthy controls. There was no attempt to match groups regarding characteristics such as age and sex, which may affect body perception. However, when assessing the difference between groups an ANCOVA was used which adjusted for age, sex and BMI. We hypothesised that people with CLBP would differ from healthy controls on the construct of self-perception of the back, as assessed by the FreBAQ-G, in that healthy people would on average score lower compared to people with CLBP, demonstrating better self-perception of the back in healthy controls.

## Results

### Translation and face validity

The majority of participants in both groups found the FreBAQ-G to be a complete and comprehensible measure, which could be completed within an appropriate period of time (Table 1).

**Table 1:** Results of feedback form.

Item		CLBP group (n = 35)	Control group (n = 48)
1	Completeness of Contents N (%)	25 (71.4)	39 (81.3)
2	Comprehensibility N (%)	29 (82.9)	37 (77.1)
3	Appropriateness of time to complete Mean (SD) (0–10 scoring system)	9.9 (0.8)	9.9 (0.2)

N = proportion of participants stating complete agreement or acceptance with item; CLBP = chronic low back pain

In the patient group, additional questions about content covering aspects of night sleep, stair climbing, current awareness of posture, morning stiffness and current sensory abnormalities hampering body awareness were suggested for inclusion. Regarding comprehensibility it was stated by one patient that the double negative expressions in question 4, 5, and 6 could be misleading. With respect to questions 2 and 3 one individual suggested to provide examples of which specific activities were meant.

Given the qualitative feedback, and as all three scores of the feedback form were well below the preset threshold of 50% negative responses, it was judged that the translation process revealed no obvious cultural adaptations necessary for a German speaking population.

### Participant characteristics and questionnaire responses

The participant characteristics for each group are shown in Table 2. On average the control group was 16 years younger and there were small differences in sex and BMI between groups. Thus age, sex and BMI were adjusted for as covariates in the comparison between groups.

**Table 2:** Descriptive statistics of study sample.

	CLBP patients (n = 35) Mean (SD^a^) or N (%)	Healthy Controls (n = 48) Mean (SD) or N (%)	p-values
**Study sample demographic information**			
Gender (female)	23 (65.8%)	30 (62.5%)	p = 0.76
Age (years)	52 (15.2)	36 (17.5)	**p<0.01**
Height (cm)	172 (9.9)	173 (8.3)	p = 0.66
Weight (kg)	76 (17.1)	69 (11.8)	p = 0.16
**BMI**	25.3 (4.9)	23.1 (2.6)	p = 0.09
**Work Status**			
Employed or student	20 (54.3%)	43 (89.6%)	
Retired (age related)	12 (34.3%)	5 (10.4%)	
Certified sick due to back pain	3 (8.6%)	0 (0%)	
Certified sick due to other reasons	1 (2.9%)	0 (0%)	
**Clinical Status**			
Symptom Duration (years)	11 (11)		
**BPI T1**:			
Pain Severity	3.6 (2.0)		
Pain Interference	2.6 (2.0)		
**BPI T2:**			
Pain Severity	3.5 (1.9)		
Pain Interference	2.1 (1.8)		
**RMDQ**	7.5 (4.6)		
**HADS:**			
Anxiety	5.2 (3.4)	4.8 (2.2)	p = 0.08
Depression	4.5 (3.1)	2.3 (1.9)	**p<0.01**
**EuroQuol:**			
VAS (%)	65 (21)		
Index Value	0.81 (0.20)		

SD = Standard Deviation, BMI = Body Mass index, BPI = Brief Pain Inventory, T1 = Session 1, T2 = Session 2, RMDQ = Roland Morris Disability Questionnaire, HADS = Hospital Anxiety and Depression Scale; EuroQuol = Euroquol 5D-3L; VAS = Visual analogue scale; **bold figures indicate statistical significance on 0.05 level.**

For the patient group, pain severity at time 1 and 2, were 3.6 and 3.5 respectively, defined as mild severity [50]. The average back related physical function was 7.5 at time 1, defined as a mild-to-moderate functional impairment [51]. In addition, both groups had similar HADS anxiety scores (mean (SD) CLBP group: 5.2 (3.4), control group: 4.8 (2.2)), both of which could be interpreted as normal [52].

Of the 35 participants with CLBP, two did not answer item 2 at timepoint one, one did not answer item 8 at timepoint one and two and one participant did not answer item 7 at timepoint two. In all, missing items account for 1.6% of the data, thus it is unlikely that they had a significant impact on the overall results.

The frequencies of FreBAQ-G responses, as well as the mean and median scores for the patient group are displayed per item in Table 3. All nine items were at least endorsed at some level, although reported frequencies differed across items. Items 2 and 9 were the most often endorsed. In contrast, items 6, 7 and 8 were the most infrequently endorsed, with more than 80% of participants stating that their back never feels shrunk (item 8). In contrast, items 3, 4, 5 and 6 were not endorsed on the upper end of the Likert scale (‘always feels like this’).

**Table 3:** Frequency of responses to each FreBAQ-G item in the patient group (n = 35).

Item	Response category	Never feels like that	Rarely feels like that	Occasionally or some of the time feels like that	Often, or a moderate amount of time feels like that	Always, or most of the time feels like that	Median	Mean
	Scoring	0	1	2	3	4		
	Frequency of Responses	N (%)	N (%)	N (%)	N (%)	N (%)		
1	My back feels as though it is not part of the rest of my body	19 (54.3)	6 (17.1)	4 (11.4)	5 (14.3)	1 (2.9)	0	0.9
2	I need to focus all my attention on my back to make it move the way I want it to	7 (20.0)	9 (25.7)	8 (22.9)	9 (25.7)	2 (5.7)	2	1.6
3	I feel as if my back sometimes moves involuntarily, without my control	15 (42.9)	10 (28.6)	8 (22.9)	2 (5.7)	0 (0)	1	0.9
4	When performing everyday tasks, I don’t know how my back is moving	13 (37.1)	11 (31.4)	8 (22.9)	3 (8.6)	0 (0)	1	1.0
5	I am not sure exactly what position my back is in	15 (42.9)	9 (25.7)	8 (22.9)	3 (8.6)	0 (0)	1	1.0
6	I can’t perceive the exact outline of my back	20 (57.1)	7 (20.0)	5 (14.3)	3 (8.6)	0 (0)	0	0.7
7	My back feels like it is enlarged (swollen)	23 (65.7)	1 (2.9)	7 (20.0)	3 (8.6)	1 (2.9)	0	0.8
8	My back feels like it has shrunk	28 (80.0)	3 (8.6)	2 (5.7)	1 (2.9)	1 (2.9)	0	0.3
9	My back feels lopsided (asymmetrical)	12 (34.3)	5 (14.3)	6 (17.1)	7 (20.0)	5 (14.3)	2	1.7

N = absolute frequency responses, FreBAQ-G = Fremantle Back Awareness Questionnaire German

### Relationship to clinical status

The associations between the FreBAQ-G and the clinical characteristics in the patient group were moderate for all characteristics except for duration of symptoms, which was unrelated to the FreBAQ-G (see Table 4).

**Table 4:** Univariate correlations between FreBAQ-G total scores and clinical characteristics in the patient population (n = 35).

	Correlation	p-value
Anxiety (HADS-A)^a^	0.37	0.03*
Depression (HADS-D)^b^	0.52	<0.001*
Duration of Symptoms (Years) ^b^	0.06	0.72
Pain severity (BPI) ^a^	0.32	0.07
Pain interference (BPI)^b^	0.47	<0.001*
Back related disability (RMDQ^b^	0.46	<0.001*

HADS-A = Hospital Anxiety and Depression Scale- Anxiety; HADS-D = Hospital Anxiety and Depression Scale-Depression; BPI = Brief Pain Inventory; RMDQ = Roland-Morris-Disability Questionnaire.

* Correlation is significant at the 0.05 level (2-tailed).

^a^Pearson correlation coefficient.

^b^Spearman correlation coefficient.

### Reliability

#### Intra-observer reliability

**Fig 1:**
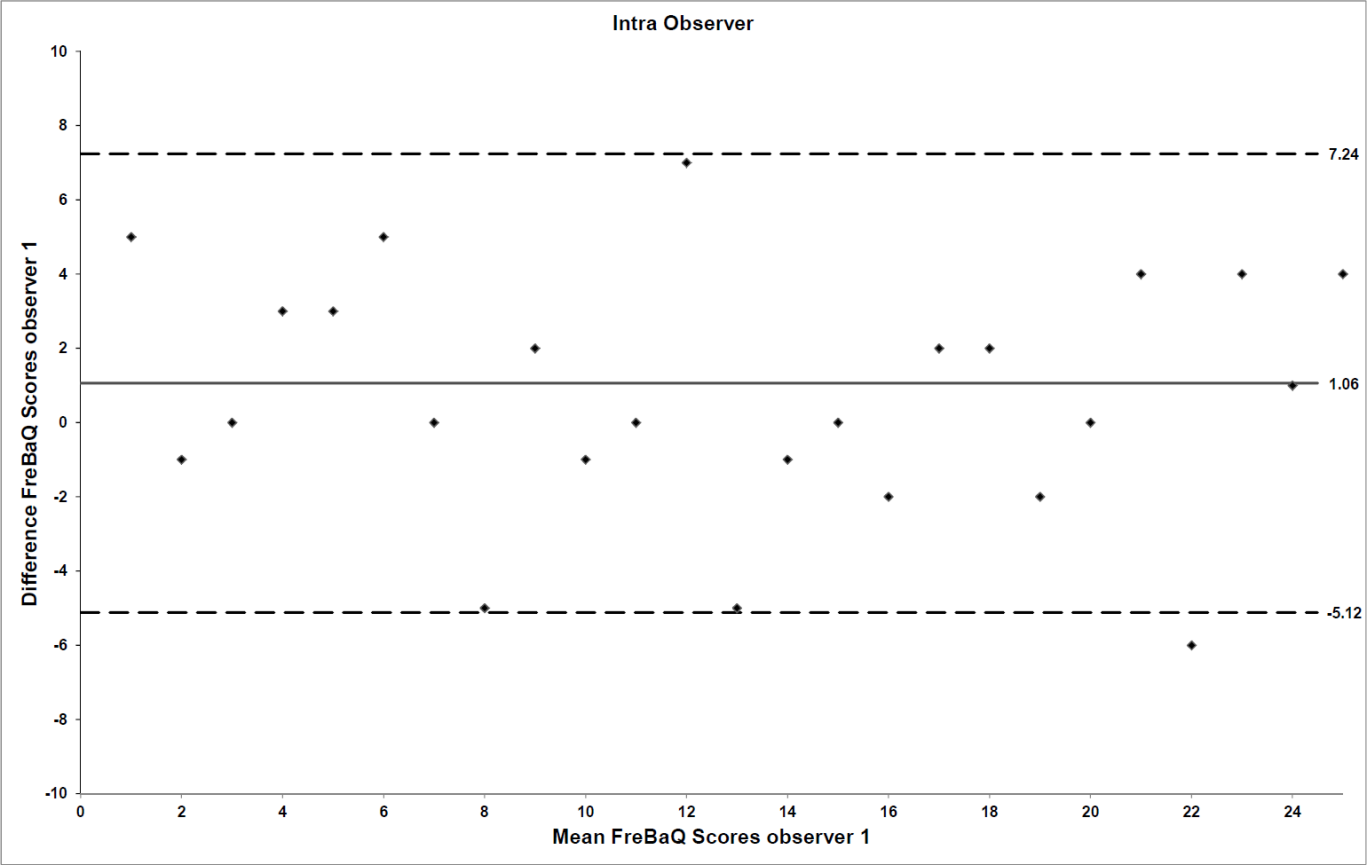
Limits of agreement for intra-observer reliability. For intra-observer reliability, the FreBAQ-G difference scores for assessor 1 at day 1 and 2 are plotted against their mean scores. Mean session differences (systematic bias) are displayed by solid lines and limits of agreement by dashed lines.

The mean value for the FreBAQ-G scores obtained from the participants by assessor 1 on day 1 was 8.8 (SD 6.1) and 7.8 (SD 7.0) on day 2. The mean FreBAQ-G difference score within one week for assessor 1 was 1.06 (95% CI: -0.03 to 2.14, p = 0.055). The ICC_3.1_ values for absolute agreement and consistency were 0.88 (95%CI: 0.77 to 0.94) and 0.89 (95%CI: 0.79 to 0.94), respectively. The Bland and Altman plot for the individual differences between day 1 and 2 for assessor 1 is shown in Fig 1.

#### Inter-observer reliability

**Fig 2:**
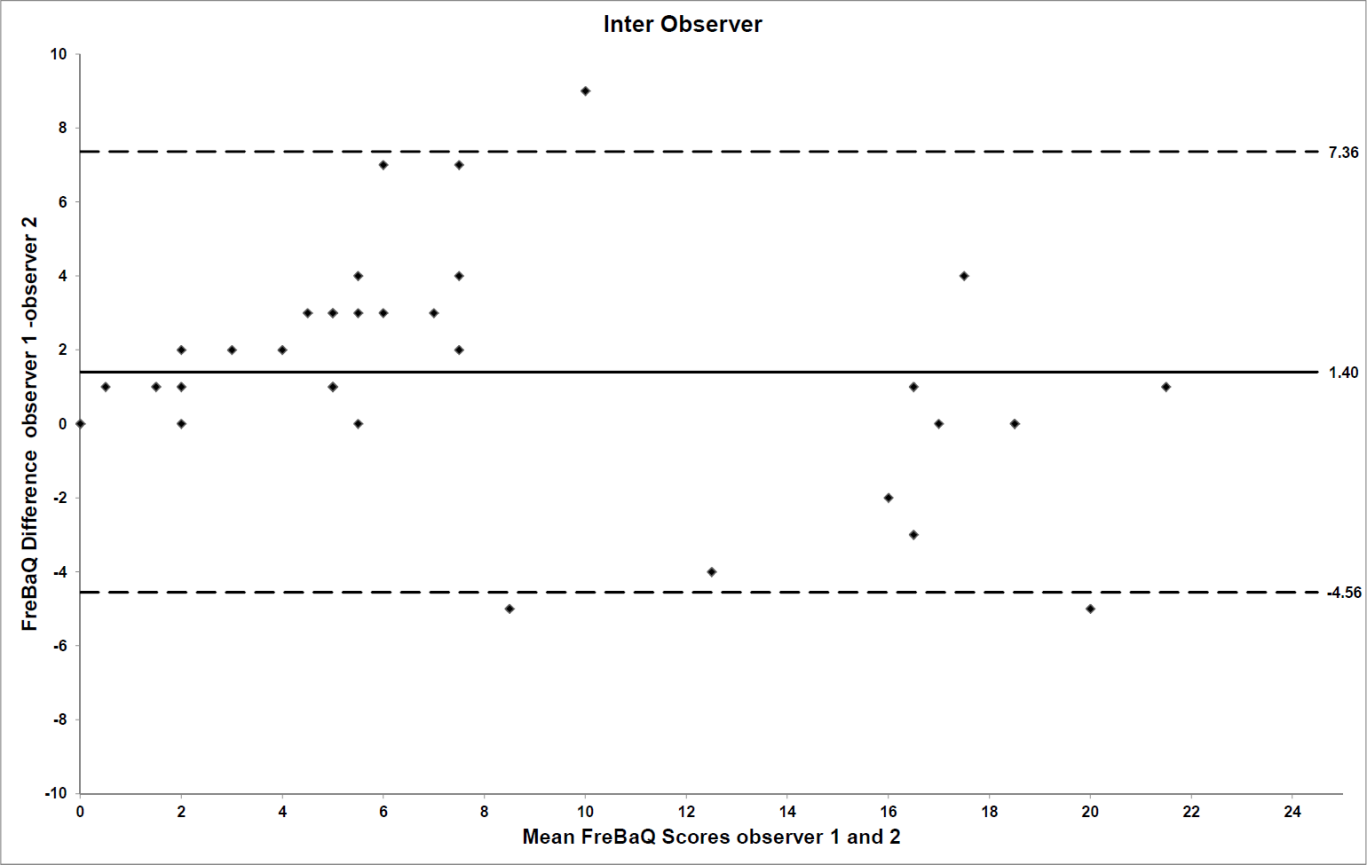
Limits of agreement for inter-observer reliability. For inter-observer reliability, the FreBAQ-G difference scores for assessor 1 and 2 are plotted against their mean scores. Mean session differences (systematic bias) are displayed by solid lines and limits of agreement by dashed lines.

The mean value for the FreBAQ-G scores obtained from the participants by assessor 1 and 2 on day 1 was 8.8 (SD 6.1) and 7.4 (SD 7.2) respectively. The mean FreBAQ-G difference score on the same day between assessors 1 and 2 was 1.4 (95% CI: 0.36 to 2.45, p = 0.01).The ICC_3.1_ values for absolute agreement and consistency were 0.88 (95%CI: 0.75 to 0.94) and 0.90 (95%CI: 0.81 to 0.95), respectively. The Bland and Altman plot for the individual differences between assessor 1 and 2 is shown in Fig 2.

The systematic bias for inter- and intra-observer reliability in both cases equaled approximately one unit on the 0–36 FreBAQ-G scale. All data quantifying the systematic and random error components of the reliability analysis are displayed in Table 5.

**Table 5:** Intra–and Inter-observer reliability data.

	Intra-observer	Inter-observer
Mean session difference	**1.06 (-0.03–2.14)**	**1.40 (0.36–2.44)**
SD of session differences	**3.15 (2.55–4.13)**	**3.04 (2.46–3.98)**
Within-subjects SD (SEM)	**2.23 (1.80–2.92)**	**2.15 (1.74–2.82)**
Coefficient of variation (%)	**26.86 (21.71–35.16)**	**26.45 (21.39–34.65)**
Limits of agreement (LOA)	**6.18 (-5.12–7.24)**	**5.96 (-4.56–7.36)**
ICC_3.1 absolute agreement_ (95%CI)	**0.88 (0.75–0.94)**	**0.90 (0.79–0.94)**

SD = Standard Deviation; SEM = Standard Error of Measurement; CI = Confidence Interval, ICC_3.1_ = Intraclass Correlation coefficient (absolute agreement). Ninety-five percent confidence intervals are presented in brackets for all metrics.

### Validity

#### Internal consistency

A Cronbach’s alpha value of 0.91 indicated an adequate internal consistency of all items of the German version of the FreBAQ in people with chronic low back pain. Table 6 displays Cronbach’s Alpha values, given that one out of nine items was deleted, as well as inter-item correlations and total-item correlations.

**Table 6:** Internal consistency of the German Fremantle back awareness questionnaire in people with chronic low back pain.

FreBAQ-G item	Cronbach’s Alpha if item deleted	Item-total Correlation	Inter-Item Correlation matrix								
			Item 1	Item 2	Item 3	Item 4	Item 5	Item 6	Item 7	Item 8	Item 9
Item 1	0.90	0.74		0.66	0.53	0.59	0.67	0.72	0.47	0.30	0.52
Item 2	0.90	0.71	0.66		0.75	0.60	0.64	0.64	0.47	0.15	0.42
Item 3	0.90	0.76	0.53	0.75		0.64	0.68	0.75	0.45	0.38	0.45
Item 4	0.90	0.73	0.59	0.60	0.64		0.70	0.64	0.47	0.27	0.52
Item 5	0.89	0.84	0.67	0.64	0.68	0.70		0.77	0.45	0.62	0.59
Item 6	0.89	0.81	0.72	0.64	0.75	0.64	0.77		0.42	0.52	0.54
Item 7	0.91	0.57	0.47	0.47	0.45	0.47	0.45	0.42		0.08	0.63
Item 8	0.91	0.43	0.30	0.15	0.38	0.27	0.62	0.52	0.08		0.38
Item 9	0.90	0.66	0.52	0.42	0.45	0.52	0.59	0.54	0.63	0.38	

FreBAQ-G = Fremantle Back Awareness Questionnaire-German version

Moreover, the internal consistency score was not severely affected by deletion of one item and correlations greater 0.7 were found between each item and the total score except for item 7 (r = 0.57), item 8 (r = 0.43) and item 9 (0.66).

#### Known-groups validity

The FreBAQ-G total scores in the patient group ranged from 0–21, the mean score (SD) was 8.8 (6.1) and the median score 7.0. In the control group, the total FreBAQ-G score ranged from 0–13, the mean score (SD) was 4.0 (3.3) and the median score was 3.0. FreBAQ-G scores were, on average, higher in the CLBP group compared to the control group [unadjusted mean difference (95%CI) 4.8 (2.55 to 7.15), p<0.01].

There was a statistically significant effect between groups regarding the FreBAQ-G scores after adjusting for age, gender and BMI (F (1.78) = 20.39, p<0.001, adjusted R^2^ = 0.22). The adjusted mean scores for the patient group was 9.1 (95%CI: 7.77 to 10.87) and 3.7 (95%CI: 2.31 to 5.18) for the control group with an adjusted mean difference of 5.4 (95%CI: 3.02 to 7.79, p<0.01).

#### Convergent validity

**Fig 3:**
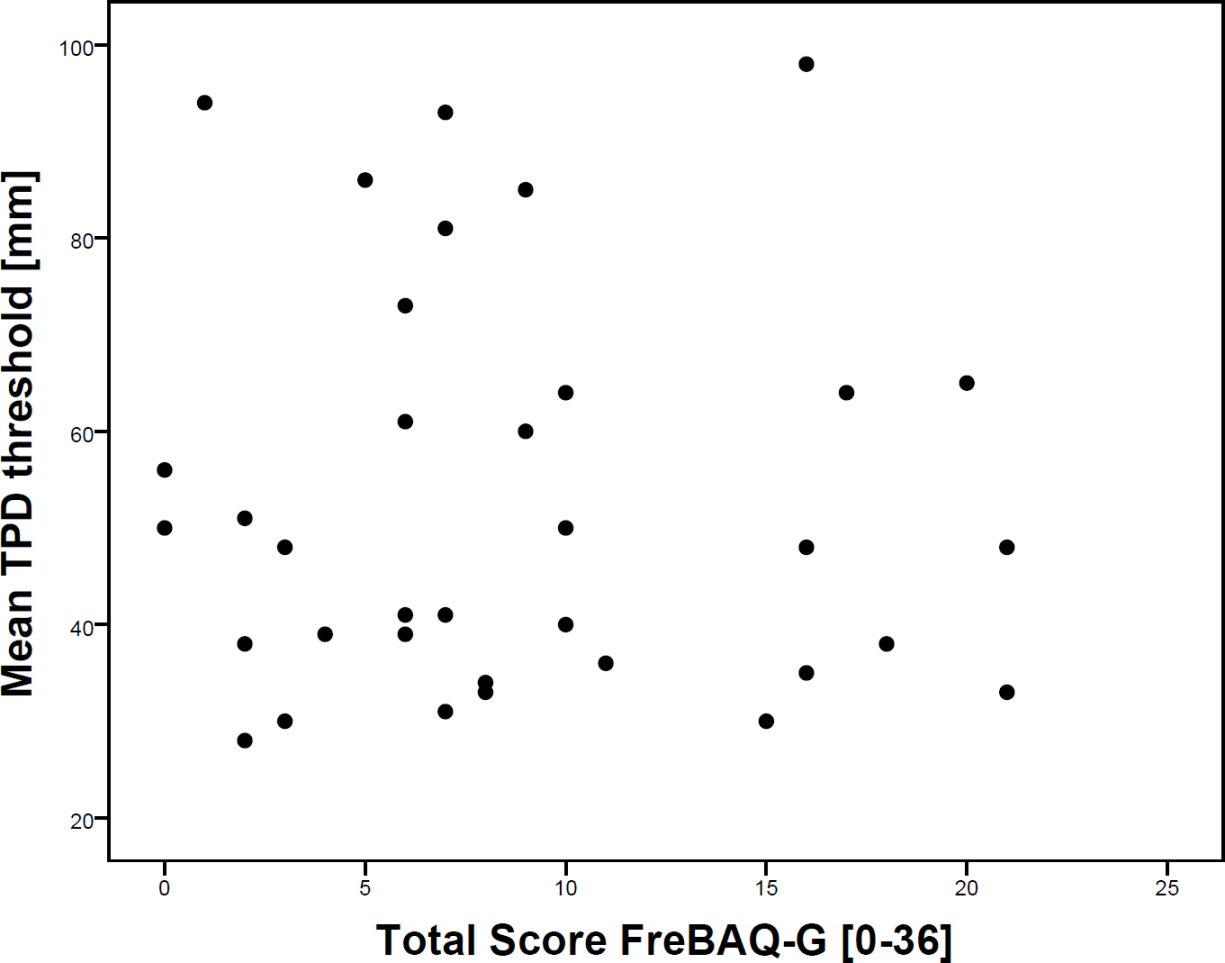
Scatterplot of the total FreBAQ-G score plotted against the mean TPD thresholds for the patient group.

The total FreBAQ scores were not associated with the mean TPD thresholds (Spearman’s *rho* = -0.05, p = 0.79) in the patient group (see Fig 3).

## Discussion

Participants found the FreBAQ-G demonstrated completeness of contents, comprehensibility and could be completed within an acceptable amount of time. These findings are in line with the results of the cross-cultural adaptation of the Dutch version of the FreBAQ-Q [28], in which participants (n = 22) with CLBP reported an overall acceptable comprehensibility of 77% and an acceptable level of completeness of contents of 82%. Quantitatively, the mean score of 8.8 for the patients on the FreBAQ-G, was similar to those reported for the original English version (10.8) [26] and the Dutch version (11) [28]. This adds confidence to the translation process and cross-cultural validity of the FreBAQ-G. Three out of 35 participants in the patient group scored 0 in total, equaling 9% of the total scores. This was below our predefined criteria of 15%, suggesting that floor/ceiling effects were not an issue for the questionnaire as a total score. However, from the frequency of responses per item potential floor effects of the FreBAQ-G could be deduced while there was no evidence of ceiling effects. These item specific floor effects could have artificially enhanced the level of reliability and whilst have a detrimental effect on the validity of the FreBAQ-G reported in this study.

The FreBAQ-G demonstrated adequate internal consistency, with a Cronbach’s alpha of 0.91 being slightly higher compared to other translated versions [27, 28]. However, as our sample size was smaller than those of the other validation studies, these results need to be interpreted cautiously.

The FreBAQ-G showed moderate intra and inter-observer reliability with ICC_3.1_ values of **≥**0.88. These values were similar (or higher) to those reported for the Japanese [27] (ICC_3,1_ of 0.81 (95% CI: 0.67–0.89)), Dutch [28] (ICC_2.1_ = 0.69 (95%CI: (0.51–0.82)) and original English version [26] (ICC_2.1_ = 0.65 (95% CI: 0.307–0.848)). A systematic bias of one unit between time 1 and 2 for observer 1 indicated some small learning effects, thus a familiarization session may be warranted when using this questionnaire.

The SEM (intra- and inter-observer) in our study was ~2units, below the SEM of 3.5 reported by Janssens et al [28]. However, with 95% limits of agreement ~6units, this indicates an individual patient with CLBP could change by as much as 6units due to normal variation. In addition, a random error component of ~26% (CV) suggests the FreBAQ-G may be more appropriately used on a group level rather than an individual patient level.

To understand if the FreBAQ-G has sufficient reliability for research purposes it can be useful to use the estimated variability of the measure and its minimally clinically important difference (MCID) to calculate sample sizes for different study designs. There is no existing empirically derived MCID for the FreBAQ. Using 0.5 of a standard deviation as a clinically worthwhile change one could estimate an MCID of approximately 3.0 units for power calculation purposes. Assuming the SD of change is 3.15 (see Table 5) it can be estimated that n = 14 would be required for a single arm pre-post study (two-tailed significance level < 0.05, statistical power = 90%) to detect the difference between a null hypothesis mean of 0.0 and an alternative mean of 3.0 units. Within an RCT design, under the same conditions, a sample size of n = 25 in each arm would be required. Both estimated sample sizes could be considered achievable within a musculoskeletal research context, supporting the potential of the FreBAQ in research.

The convergent validity of the FreBAQ-G was assessed by correlating it with TPD. There was no correlation between the FreBAQ-G and the TPD, in contrast to our initial hypothesis. These results were in keeping with Wand et al [29] who found no correlation between the English version of the FreBAQ and TPD in a sample of 34 pregnant women. This questions the assumption whether both assessments measure the same construct, although previous studies have demonstrated a relationship between body image drawings and tactile acuity in patients with CLBP [13, 14]. However, outlining or drawing one’s perceived body image and answering dedicated questions regarding one’s perceived body awareness might require different cognitive and self-reflective skills. Moreover, TPD testing constitutes a direct measurement requiring touch. Hence, TPD could be seen as a test to investigate peripheral innervation density and/or intact neural sensory pathways rather than a person’s perceived body image [53].

Body perception as measured by the FreBAQ-G was correlated with a number of the clinical outcomes assessed. This implies that the FreBAQ-G may have clinical utility and body perception may be a clinically relevant construct in this patient population. In our study sample, disturbed body perception was associated with pain interference scores (BPI-I), but not with symptom duration. In addition, FreBAQ-G scores showed moderate correlations to back related disability (RMDQ) and anxiety and depression scores (HADS). It may be possible that an altered self-perception of the back, in particular motor neglect aspects, might contribute to motor control impairments, resulting in higher back related disability scores [54, 55]. In addition, a growing body of evidence supports the notion that anxiety and depression negatively affect an individual’s confidence in an adequate loading of the back and might hence contribute to the distortion of the self-perception of the back [56, 57].

Our findings are partly in line with both English study samples, where statistically significant correlations to pain severity were found (Pearson’s r = 0.40, p = 0.04)[26] and (Pearson’s r = 0.27, p<0.001) [25]. The strength of the relationship between the FreBAQ-G and pain severity in our sample was similar to those studies (r = 0.32, p = 0.07). In addition, the Japanese sample [27] showed only correlations to back pain intensity in motion whereas the Dutch sample did not demonstrate any correlations to pain intensity at all [28]. Differences between our findings and those of other studies may due to differences in methodology. The differences here could be attributable to the greater anxiety and depression scores in our study sample compared to the Japanese study and that our sample showed higher values in pain scores interfering with daily function (BPI-pain interference scores) in contrast to the pain intensity in motion scores in the Japanese sample. However, all existing versions of the FreBAQ showed a correlation between back related disability and disturbed body perception [26–28]. This could be explained by the fact that an inability to adequately perform activities of daily living might be associated with reduced sensorimotor lumbopelvic control [17, 55].

In contrast to Wand et al [26] and Nishigami et al [27], our sample showed an association between anxiety and depression scores and disturbed body perception. This finding may be attributable to the notion that cognitive emotional aspects of pain drive central nervous adaptation, such as central sensitization, which may in turn modulate sensorimotor control and body perception [58].

The FreBAQ-G demonstrated a degree of known-groups validity, identifying a difference of ~5units between individuals with CLBP and health participants, after adjusting for age, gender and BMI. The difference between groups was half that previously reported (11.0 units) using the original FreBAQ [26]. This difference may have been due to sample differences in both the clinical and control participants between that study and our study.

### Strengths and limitations

Regarding the translation process, an initial pre-testing phase in a smaller sample of patients with CLBP could have been utilized to reveal and resolve any difficulties regarding comprehensibility and completeness of contents before commencing the study. However, patients were satisfied with all usability aspects. In addition, we did not measure the exact amount of time it took patients to complete the questionnaire though participants reported that the time to complete was appropriate in their opinion. The current version of the FreBAQ-G demonstrated evidence of floor effects on an item level. This might have adversely affected reliability and validity scores. However, regarding sum scores, the percentage of respondents scoring 0 were below the pre-defined cut-off value of 15%. Hence, our main criterion demonstrated that floor effects did not appear to be an issue in our sample.

Although all the patient participants in our study were patients accessing a health care setting for treatment of their CLBP they were on the low end of the spectrum for the range of clinical measures that were used, especially regarding anxiety and depression scores. Thus, our findings may not be generalisable to the wider CLBP population, especially those scoring higher on the clinical spectrum.

Our final sample size of 35 patients was lower than current recommendations of 40 participants or more for reliability studies [59]. Initially, 51 individuals were contacted. Ten people did not respond to any further communication and six did not meet the inclusion criteria. In addition, for the known-groups validity testing, the design would have been improved if groups were matched on key characteristics such as age, sex and BMI, however these were adjusted for statistically in the analysis.

To assess the convergent validity of the FreBAQ-G, its scores were correlated to TPD performance which claims to measure the same/or similar construct. The choice of comparator measure to assess convergent validity was difficult as there is no gold standard measure for the construct of self-perception of the back. A recent systematic review published by our group [24] found there were no existing measures of sensory motor perception that have demonstrated adequate levels of validity and reliability. However, the review did identify TPD as one of the most promising measures. In addition, TPD is one of the most commonly used measures of back perception within the literature [60–62] and it has been previously used as comparator for other measures of sensorimotor back function [17]. Thus it was chosen as the comparator in this study but the findings should be interpreted cautiously. Finally, while components of the validity of the FreBAQ-G have been assessed, definitive evidence that the FreBAQ-G measures the construct back self-perception is lacking. This is likely attributable to the fact that self-perception is a complex construct to define and, as previously stated, no definitive gold standard measure exists. Further exploration of the validity of the FreBAQ-G is warranted.

### Clinical implications

The translation and assessment of the German FreBAQ is an important step in the use of this questionnaire in people with CLBP, as is makes it available to a German speaking population of 118 million people [63]. The FreBAQ-G constitutes a time efficient, low-cost and safe assessment tool, provisionally demonstrating acceptable levels of reliability for research purposes though it is unclear if the level of reliability is sufficient to be used at the individual patient level. There is evidence of small learning effects, thus a familiarisation session would appear warranted.

The FreBAQ-G is not proposed as an alternative outcome measure to established clinical measures such as pain and function. However, if a researcher/clinician wishes to assess the specific construct of self-perception of the back very few instruments are available and the clinimetric properties of those measures are limited [24]. If self-perception of the back is a construct of interest the FreBAQ-G could be a potentially useful tool. However, it should be employed knowing that the current level of validity is unclear and its level of reliability is not yet sufficient to be used on an individual patient level. Further research is required before firm recommendations on the use of the FreBAQ-G can be made.

## Conclusion

### Main results

We created a German translation of the FreBAQ. The FreBAQ-G demonstrated a degree of reliability and known-groups validity, while it did not demonstrate convergent validity against a measure, which purports to assess the same construct. These findings are broadly in keeping with other language versions of the questionnaire. The clinimetric properties of the FreBAQ-G require further investigation as a simple measure of self-perception of the back.

### Practical tips

Given the degree of measurement error the FreBAQ-G could potentially be employed for research purposes to assess back self-perception but it may be too variable to monitor change in individual patients. To minimize learning effects, a familiarisation trial should be considered. The validity of the FreBAQ-G requires further exploration.

## Supporting information

S1 File The Fremantle Back Awareness Questionnaire-German (FreBAQ-G). (PDF)

S1 Table Supporting Information–Raw Data set. FreBAQ = Fremantle Back Awareness Questionnaire; TPD = Two-Point Discrimination; HC = Healthy controls.
(DOCX)
