# Supplementary material for: The translation, validity and reliability of the German version of the Fremantle Back Awareness Questionnaire
Source: PLoS One. 2018 Oct 4;13(10):e0205244. doi: 10.1371/journal.pone.0205244 (PMC6171905; doi:10.1371/journal.pone.0205244)
Supplement: S1 File — (PDF) [file pone.0205244.s001.pdf]

S1 Appendix: The Fremantle Back Awareness Questionnaire-German (FreBAQ-G)

Teesside University is sponsoring this project for the purposes of research governance

Der Fremantle Fragebogen zur Wahrnehmung des Rückens

Name, Vorname	
Geburtsdatum	
Datum	

Hier finden Sie eine Auswahl von Aussagen anderer Patienten, die beschreiben, wie diese ihren Rücken wahrnehmen. Bitte nutzen Sie die folgende Abstufungsskala, um anzugeben, inwieweit Sie Ihren Rücken, **in dem Moment, wo Sie Schmerzen haben,** in ähnlicher Weise wahrnehmen.

0 = Fühlt sich nie so an

1 = Fühlt sich selten so an

2 = Fühlt sich gelegentlich bzw. manchmal so an

3 = Fühlt sich häufig bzw. oft so an

4 = Fühlt sich immer bzw. meistens so an

	Fühlt sich nie so an	Fühlt sich selten so an	Fühlt sich gelegentlich bzw. manchmal so an	Fühlt sich häufig bzw. oft so an	Fühlt sich immer bzw. meistens so an
Mein Rücken fühlt sich so an, als würde er nicht mehr zum restlichen Teil meines Körpers gehören.	0	1	2	3	4
Ich muss meine ganze Aufmerksamkeit auf meinen Rücken richten, damit er sich so bewegt, wie ich es will.	0	1	2	3	4
Manchmal habe ich keine Kontrolle über die Bewegungen, die mein Rücken macht.	0	1	2	3	4
Wenn ich Alltagsaufgaben ausführe, weiß ich **nicht,** wie mein Rücken sich bewegt.	0	1	2	3	4
Wenn ich Alltagsaufgaben ausführe, bin ich mir nicht genau sicher, in welcher Position sich mein Rücken befindet.	0	1	2	3	4
Ich kann die Umrisse meines Rückens nicht genau wahrnehmen.	0	1	2	3	4
Mein Rücken fühlt sich so an, als sei er größer als er eigentlich ist (geschwollen).	0	1	2	3	4
Mein Rücken fühlt sich so an, als sei er kleiner geworden.	0	1	2	3	4
Mein Rücken fühlt sich so an, als sei er schief (asymmetrisch).	0	1	2	3	4
