# Supplementary material for: The translation, validity and reliability of the German version of the Fremantle Back Awareness Questionnaire
Source: PLoS One. 2018 Oct 4;13(10):e0205244. doi: 10.1371/journal.pone.0205244 (PMC6171905; doi:10.1371/journal.pone.0205244)
Supplement: S1 Table — FreBAQ = Fremantle Back Awareness Questionnaire; TPD = Two-Point Discrimination; HC = Healthy controls. (DOCX) [file pone.0205244.s002.docx]

**S1 Table. Supporting Information – Raw Data set**

| Participant Number | FreBAQ total scores session 1 - Assessor 1 | FreBAQ total scores session 1 -Assessor 2 | FreBAQ total scores session 2 -Assessor 1 | Mean of 2-5 TPD measurements (mm) | Participant Number | FreBAQ total scores HC |
| --- | --- | --- | --- | --- | --- | --- |
| CLBP 1 | 8 | 4 | 3 | 34 | HC 1 | 3 |
| CLBP 2 | 16 | 19 | 17 | 35 | HC 2 | 5 |
| CLBP 3 | 2 | 2 | 2 | 38 | HC 3 | 2 |
| CLBP 4 | 20 | 20 | 17 | 65 | HC 4 | 5 |
| CLBP 5 | 6 | 3 | 3 | 39 | HC 5 | 1 |
| CLBP 6 | 10 | 6 | 5 | 64 | HC 6 | 0 |
| CLBP 7 | 0 | 0 | 0 | 56 | HC 7 | 3 |
| CLBP 8 | 10 | 14 | 15 | 40 | HC 8 | 7 |
| CLBP 9 | 11 | 2 | 9 | 36 | HC 9 | 4 |
| CLBP 10 | 8 | 13 | 9 | 33 | HC 10 | 9 |
| CLBP 11 | 5 | 4 | 5 | 86 | HC 11 | 7 |
| CLBP 12 | 21 | 17 | 14 | 48 | HC 12 | 5 |
| CLBP 13 | 16 | 16 | 21 | 48 | HC 13 | 2 |
| CLBP 14 | 21 | 20 | 22 | 33 | HC 14 | 6 |
| CLBP 15 | 3 | 1 | 3 | 48 | HC 15 | 10 |
| CLBP 16 | 4 | 3 | 6 | 39 | HC 16 | 2 |
| CLBP 17 | 3 | 1 | 1 | 30 | HC 17 | 3 |
| CLBP 18 | 7 | 4 | 5 | 93 | HC 18 | 7 |
| CLBP 19 | 16 | 16 | 18 | 98 | HC 19 | 4 |
| CLBP 20 | 2 | 1 | 2 | 51 | HC 20 | 3 |
| CLBP 21 | 18 | 20 | 14 | 38 | HC 21 | 10 |
| CLBP 22 | 17 | 22 | 23 | 64 | HC 22 | 0 |
| CLBP 23 | 9 | 6 | 5 | 85 | HC 23 | 1 |
| CLBP 24 | 6 | 6 | 5 | 61 | HC 24 | 0 |
| CLBP 25 | 6 | 4 | 2 | 73 | HC 25 | 7 |
| CLBP 26 | 7 | 4 | 4 | 81 | HC 26 | 11 |
| CLBP 27 | 7 | 5 | 8 | 41 | HC 27 | 3 |
| CLBP 28 | 0 | 0 | 0 | 50 | HC 28 | 3 |
| CLBP 29 | 15 | 14 | 18 | 30 | HC 29 | 4 |
| CLBP 30 | 10 | 3 | 5 | 50 | HC 30 | 2 |
| CLBP 31 | 9 | 2 | 3 | 60 | HC 31 | 1 |
| CLBP 32 | 6 | 3 | 4 | 41 | HC 32 | 11 |
| CLBP 33 | 1 | 0 | 0 | 94 | HC 33 | 1 |
| CLBP 34 | 7 | 4 | 3 | 31 | HC 34 | 5 |
| CLBP 35 | 2 | 1 | 1 | 28 | HC 35 | 1 |
|  |  |  |  |  | HC 36 | 13 |
|  |  |  |  |  | HC 37 | 3 |
|  |  |  |  |  | HC 38 | 1 |
|  |  |  |  |  | HC 39 | 2 |
|  |  |  |  |  | HC 40 | 0 |
|  |  |  |  |  | HC 41 | 0 |
|  |  |  |  |  | HC 42 | 2 |
|  |  |  |  |  | HC 43 | 3 |
|  |  |  |  |  | HC 44 | 9 |
|  |  |  |  |  | HC 45 | 2 |
|  |  |  |  |  | HC 46 | 3 |
|  |  |  |  |  | HC 47 | 4 |
|  |  |  |  |  | HC 48 | 1 |
| FreBAQ=Fremantle Back Awareness Questionnaire; TPD= Two-Point Discrimination; HC=Healthy controls | | | | | | |
